# A case report of ventricular suicide following transcatheter aortic valve replacement for severe aortic stenosis in a patient with hypertrophic cardiomyopathy: the danger of abrupt afterload reduction

**DOI:** 10.1186/s43044-025-00650-2

**Published:** 2025-06-01

**Authors:** Kirtivardhan Vashistha, Akshat Banga, Ramzi Khalil, Jian Hu, Pietro Bajona, Jennifer Keeley, Srinivas Murali, Craig Alpert, Robert Biederman, Victor Farah, Vinh Nguyen

**Affiliations:** 1 Mount Sinai Morningside, New York, USA; 2 Mount Auburn Hospital, Cambridge, USA; 3 Allegheny General Hospital, Pittsburgh, USA

**Keywords:** Hypertrophic cardiomyopathy, Left ventricular outflow tract obstruction, TAVR, Aortic stenosis, Ventricular suicide

## Abstract

**Background:**

We describe a patient with severe aortic stenosis (AS) developing obstructive shock immediately following transcatheter aortic valve replacement (TAVR) secondary to a unique phenomenon termed “ventricular suicide.” Abrupt withdrawal of chronically high afterload may lead to mid-ventricular systolic collapse ± left ventricular outflow tract (LVOT) obstruction in the setting of hyperdynamic contractility, as seen in hypertrophic cardiomyopathy (HCM).

**Case presentation:**

An 88-year-old male with severe symptomatic AS presented with worsening dyspnea. Given his high surgical risk and frailty, he underwent TAVR. The patient had a history of persistent atrial fibrillation, hypertension, hyperlipidemia, prior cerebellar stroke, and severe AS. Post-TAVR, he experienced a significant blood pressure drop, leading to shock. Investigations revealed hyperdynamic left ventricular (LV) function, cavitary obliteration, and systolic anterior motion of the mitral valve. Management included intravenous fluids and phenylephrine, which stabilized his condition. He was discharged on a beta-blocker and remained asymptomatic with a normally functioning TAVR prosthesis one month post-discharge.

**Conclusion:**

HCM and its phenocopies are associated with worse outcomes post-TAVR. Prophylactic beta-blockade and hydration may prevent hemodynamic collapse in patients with anatomic substrates for ventricular suicide.

**Supplementary Information:**

The online version contains supplementary material available at 10.1186/s43044-025-00650-2.

## Background

Transcatheter aortic valve replacement (TAVR) has become a widely accepted treatment for patients with severe symptomatic aortic stenosis (AS), especially those at high surgical risk due to advanced age or comorbidities (1). However, despite the procedural benefits, TAVR can lead to unexpected complications, including a rare and critical condition termed "ventricular suicide” (2). This phenomenon is characterized by an abrupt drop in afterload, leading to mid-ventricular systolic collapse and possibly left ventricular outflow tract (LVOT) obstruction (3), particularly in patients with hypertrophic cardiomyopathy (HCM) or HCM-like physiology (4).

HCM, a condition characterized by left ventricular (LV) hypertrophy without another causative cardiac or systemic condition, can present challenges during and after TAVR. When the chronically high afterload imposed by AS is suddenly relieved, the hyperdynamic contractile state of the hypertrophied left ventricle can precipitate severe hemodynamic instability (5). This instability is exacerbated by factors such as small ventricular cavity size, severe concentric hypertrophy, and systolic anterior motion (SAM) of the mitral valve, leading to dynamic LVOT obstruction and the potential for obstructive shock (6).

In this case report, we present an 88-year-old male with severe AS who developed obstructive cardiogenic shock and hemodynamic collapse immediately following TAVR due to "ventricular suicide" and was found to have hyperdynamic LV function, cavitary obliteration, and SAM of the mitral valve, consistent with HCM.

## Case presentation

### History of presentation

An 88-year-old male with severe symptomatic aortic stenosis (AS) presented with worsening exertional dyspnea and progressive fatigue over several weeks. On initial assessment, his vitals were: blood pressure (BP) 138/76 mmHg, heart rate (HR) 78 bpm (irregularly irregular due to atrial fibrillation), respiratory rate (RR) 18 breaths per minute, and oxygen saturation (SpO_2_) 96% on room air.

Physical examination revealed a frail-appearing male with a 3/6 harsh mid-systolic murmur at the upper right sternal border radiating to the neck. Given his high surgical risk, frailty, and worsening symptoms, he was deemed not a candidate for surgical valve replacement and was scheduled for an elective TAVR.

### Past medical history

The patient had a past medical history of persistent atrial fibrillation, hypertension, hyperlipidemia, prior cerebellar stroke, and severe AS.

### Differential diagnosis

The differential diagnosis for shock following the TAVR procedure included acute coronary syndrome, heart failure with preserved ejection fraction exacerbation, acute pulmonary embolism, and hypertrophic cardiomyopathy with acute shock from LVOT obstruction.

### Investigations

**Fig. 1:**
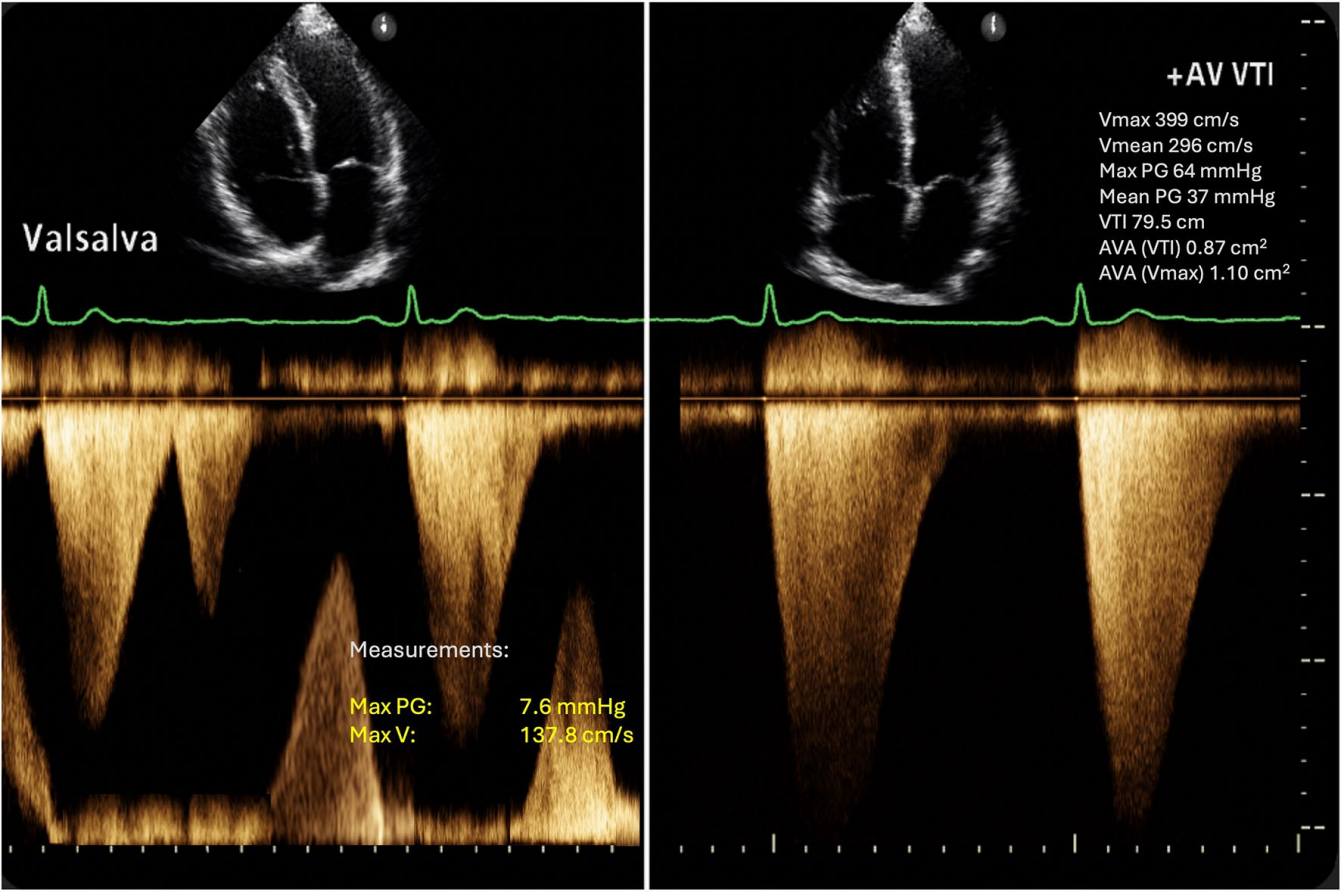
Baseline LVOT spectral Doppler showing late peaking Doppler waveform (left). Transaortic continuous Doppler indicates severe aortic stenosis by peak velocity 399 cm/s

**Fig. 2:**
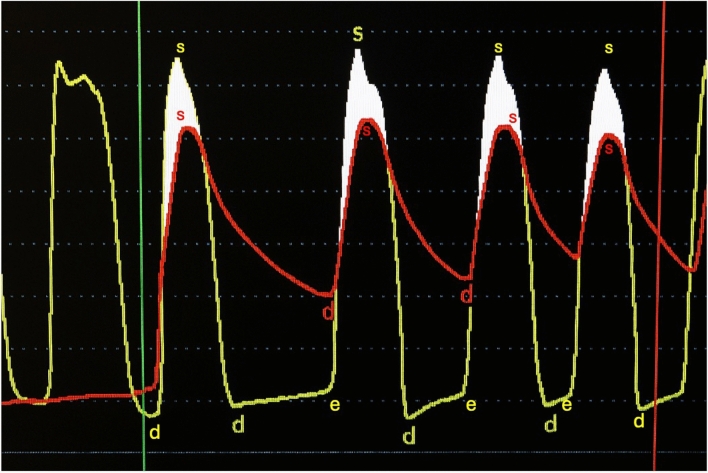
Invasive hemodynamic tracing pre-TAVR demonstrating elevated LV-Ao gradient consistent with severe aortic stenosis

Prior to undergoing the TAVR procedure, an electrocardiogram (EKG) showed chronic atrial fibrillation with a left bundle branch block. A transthoracic echocardiography (TTE) demonstrated a left ventricular ejection fraction (LVEF) of 60–64%, grade III diastolic dysfunction, severe AS with a valve area 0.9 cm^2^, mean AV gradient (AVG) 37 mmHg, AV peak velocity 3.99 m/s, and trace aortic regurgitation (Fig. 1). There was severe concentric hypertrophy (basal anteroseptal segment 19 mm; a posterior wall of 18 mm) and a small LV cavity (LVEDD 35 mm). Cardiac catheterization revealed hemodynamic parameters of AO pressure 135/73 mmHg (mean 97 mmHg), LV pressure 178/5 mmHg with a left ventricular end-diastolic pressure (LVEDP) of 14 mmHg, and a peak-to-peak gradient of 56 mmHg between the LV and AO (Fig. 2**)**. The calculated body surface area was 1.82 m^2^, with an estimated O₂ consumption of 242.3%. Computed tomography showed an Agatston valve score of 4,963, and coronary angiography demonstrated mild-to-moderate nonobstructive coronary artery disease.

**Fig. 3:**
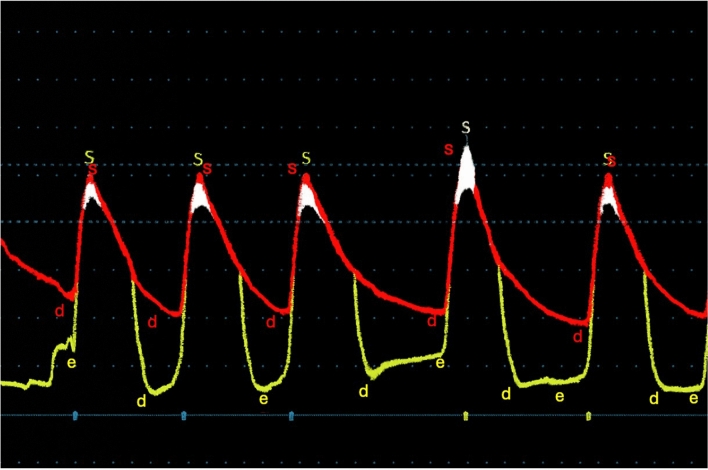
Post-TAVR Invasive LV pressure tracing showed the Brockenbrough–Braunwald–Morrow sign with a peak-to-peak pressure ~ 25 mmHg

**Fig. 4:**
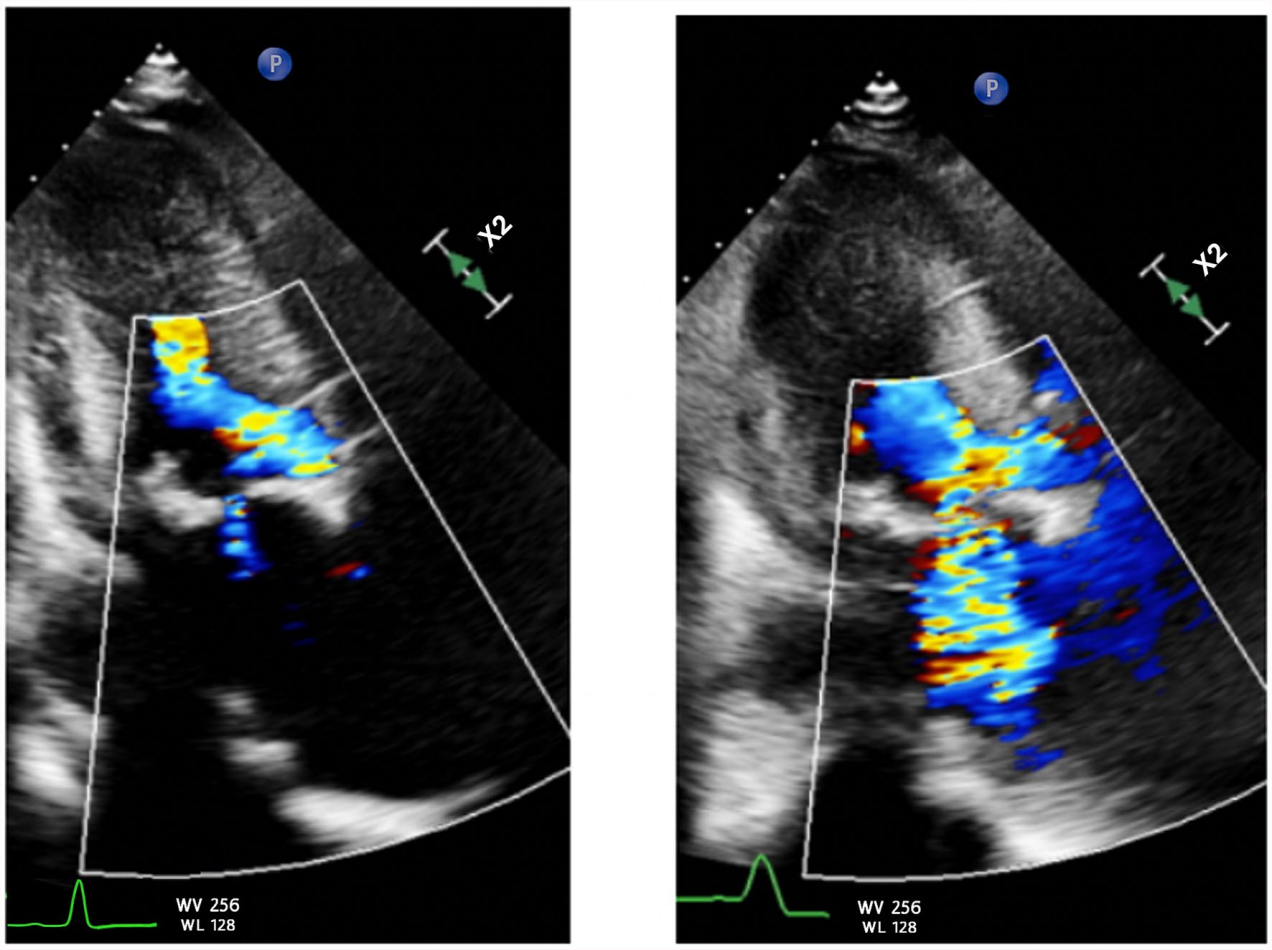
Static systolic frame showing baseline trace mitral regurgitation (left) and moderate-severe 3 + mitral regurgitation (right)

**Fig. 5:**
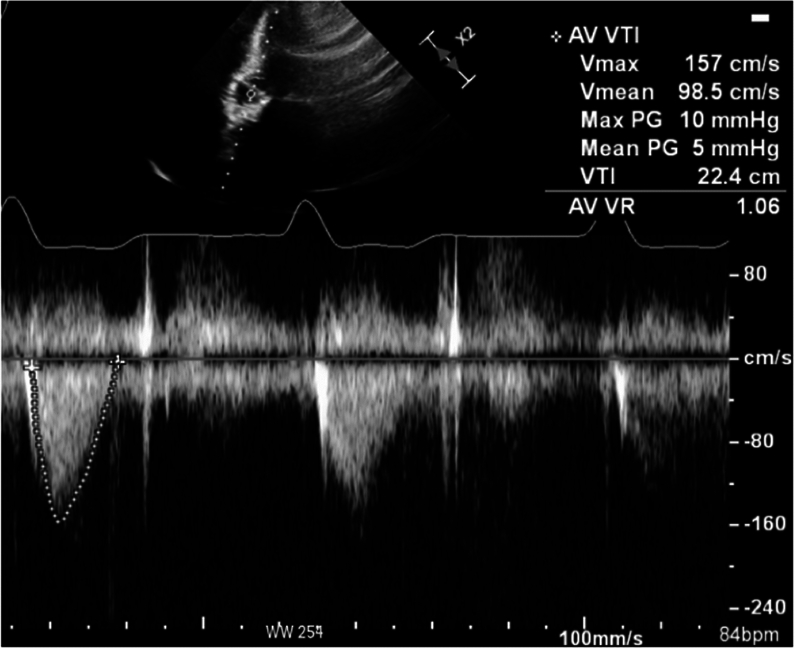
Continuous Doppler showed no significant LVOT obstruction. The peak and mean LVOT gradient were 10 mmHg and 5 mmHg, respectively

The patient underwent an elective TAVR placement with Edwards Sapien Ultra 26 mm valve. The hemodynamic profile following valve implantation showed an interval decrease in mean AVG from 56 to 6 mmHg while LVEDP remained stable around 12–14 mmHg. There was no significant gradient between the LVOT and ascending aorta (Figs. 2 and 3). Immediately following valve deployment, the patient developed hypotension with BP dropping from 135/73 mmHg to 86/42 mmHg. Heart rate increased to 120–130 bpm, and he became increasingly agitated with increased respiratory rate and worsening dyspnea. Intraprocedural echocardiography demonstrated a well-seated valve prosthesis with normal leaflet motion and no transvalvular or significant paravalvular aortic regurgitation. There was hyperdynamic LV function with cavitary obliteration, worsened mitral regurgitation, and new SAM of the mitral valve (Fig. 4, Video 1 and 2). Invasive LV pressure tracing showed the Brockenbrough–Braunwald–Morrow sign with a peak-to-peak gradient of 25 mmHg (Fig. 3). The gradient was thought to be intraventricular with no significant LVOT obstruction based on a peak LVOT gradient of 10 mmHg (Fig. 5).

Laboratory evaluation during the shock phase revealed early signs of end-organ hypoperfusion. Serum creatinine increased from 1.2 mg/dL preprocedure to 1.8 mg/dL post-TAVR. Serum lactate was elevated at 3.8 mmol/L, liver enzymes were mildly elevated, with an AST of 80 U/L and ALT of 70 U/L, and urine output decreased to 0.3 mL/kg/hr. Clinically, the patient appeared lethargic and disoriented.

To rule out coronary obstruction as a potential cause of post-TAVR hemodynamic collapse, a coronary angiogram was performed intraoperatively and demonstrated no evidence of coronary ostial compromise from valve deployment. This evaluation was preceded by careful preprocedural CT planning, which guided optimal valve sizing and confirmed adequate coronary ostial height, thereby reducing the procedural risk of coronary obstruction. Additionally, there were no dynamic ST-T changes on intraoperative ECG, and TTE revealed a hyperdynamic left ventricle with no regional wall motion abnormalities. No valvular or paravalvular regurgitation was observed. Rather, findings of SAM severe hyperdynamic ejection fraction, and intracavitary obliteration were noted, consistent with dynamic mid-ventricular obstruction rather than ischemic collapse.

### Management

Given the LV systolic collapse following acute afterload reduction, the patient was promptly managed with 1.5L of intravenous fluids and was initiated on phenylephrine infusion. His blood pressure, which had dropped to 80/50 mmHg, subsequently improved to 110/65 mmHg. Heart rate decreased from 120 to 130 bpm to 80–90 bpm, and peripheral perfusion improved.

With fluid resuscitation and vasoconstrictor support, urine output improved to > 0.5 mL/kg/hr, liver enzymes normalized, and serum lactate trended down to 1.8 mmol/L gradually. The patient was successfully weaned off phenylephrine within 24 h, maintaining stable hemodynamics and organ perfusion.

### Follow-up

He was discharged on a beta-blocker aimed at decreasing the heart rate and increasing ventricular filling time. A follow-up TTE one month later revealed a normal LVEF (55–60%), improved diastolic function (grade I), and a well-seated bioprosthetic aortic valve with normal leaflet motion. Aortic valve Doppler measurements showed a peak velocity of 2.1 m/s, mean gradient of 8 mmHg, and calculated aortic valve area (AVA) of 1.4 cm^2^. The patient remained asymptomatic with no signs of recurrent obstruction or heart failure.

## Discussion

Ventricular suicide is a rare but potentially life-threatening complication following TAVR, particularly in patients with underlying HCM or HCM-like physiology. This case report aims to emphasize the importance of preoperative risk stratification to identify patients at risk, recognizing predisposing factors, and implementing targeted management strategies to mitigate hemodynamic instability. In this case, an 88-year-old male with severe AS developed obstructive cardiogenic shock immediately following TAVR due to abrupt afterload reduction. The resultant hyperdynamic left ventricular function, cavitary obliteration, and SAM of the mitral valve led to hemodynamic collapse. Prompt recognition and hemodynamic optimization, including volume expansion, vasoconstrictor support, and heart rate modulation, are essential to preventing severe complications.

TAVR is a widely accepted treatment for severe symptomatic AS, particularly in patients with advanced age, suboptimal surgical risk, and underlying LV dysfunction (7). However, its associated complications include coronary occlusion, heart block, device embolization, LV perforation and tamponade, aortic dissection, aortic regurgitation, and ventricular suicide (8). The rare phenomenon of ventricular suicide results from acute afterload reduction in a predisposed hypertrophic LV with a small cavity, leading to cardiovascular collapse due to dynamic intraventricular gradients ± LVOT obstruction (9). Risk factors include hyperdynamic ventricular systolic function, a small, obliterated LV cavity due to hypertrophy, asymmetrical septal thickening leading to HCM-like physiology, narrowed LVOT, anterior mitral leaflet displacement, and elevated baseline LVOT gradients (10). A sudden reduction in afterload post-TAVR can precipitate a hyperdynamic state, inducing the Venturi effect on the anterior mitral leaflet and leading to acute hemodynamic collapse. Post-TAVR systolic obstruction is usually mid-ventricular due to concentric hypertrophy secondary to chronic pressure overload in AS (11). Some patients may have underlying HCM or develop HCM-like physiology due to chronic pressure overload, resulting in a septal bulge and SAM of the anterior mitral leaflet (12).

Ventricular suicide physiology is often anticipated based on the presence or absence of baseline LVOT obstruction, severe basal LV hypertrophy (LVH), a long anterior mitral valve leaflet, or evidence of SAM. Preprocedural assessment is therefore critical in identifying patients at risk. While the majority of such patients can be managed conservatively with preload optimization, beta-blockers, and nondihydropyridine calcium channel blockers (13), alcohol septal ablation (ASA) may be considered in select cases with significant baseline LVOTO. ASA, typically reserved for patients with demonstrable obstruction prior to TAVR, reduces septal thickness and LVOT gradients but carries procedural risks such as complete heart block and the need for permanent pacemaker implantation (14, 15).

In our case, the patient did not exhibit LVOTO or intraventricular gradients on preprocedural imaging. The gradient that developed post-deployment was intraventricular rather than subaortic, and thus not amenable to ASA. However, for patients who develop refractory obstructive physiology post-TAVR despite optimal medical management, urgent ASA remains a viable intervention to relieve dynamic obstruction and restore hemodynamic stability.

Treatment of ventricular suicide is aimed at increasing diastolic filling time and expanding the LV cavity. Initial management involves beta-blockers to slow the heart rate and improve ventricular filling, along with intravenous fluids to maintain preload. Vasoconstrictors such as phenylephrine are beneficial in increasing afterload and stabilizing LV end-diastolic pressure (16). Our case had an underlying conduction defect (LBBB) and may have also benefited from prophylactic transvenous pacing post-TAVR to mitigate LVOT gradients.

HCM is strongly associated with senile calcific AS and has been linked to higher in-hospital mortality and post-TAVR complications (17). Olsen et al. reported a case of hemodynamic collapse in a 56-year-old male with undiagnosed HCM undergoing TAVR, where intraoperative transesophageal echocardiography revealed SAM of the anterior mitral valve leaflet and asymmetric septal hypertrophy. Similar to our case, the patient experienced acute hemodynamic deterioration following valve deployment, attributed to unrecognized dynamic obstruction, though specific details of end-organ hypoperfusion were not reported. They noted that in patients with severe AS, the high LV afterload and arterial impedance often mask subvalvular gradients, making the condition difficult to detect preoperatively (18). Another case by Suh et al. described an 82-year-old woman undergoing TAVR who developed obstructive shock due to SAM and asymmetric septal hypertrophy (29). Both cases share key physiologic features with our patient, including septal hypertrophy, hyperdynamic left ventricular function, and SAM-induced obstruction, and like ours, they were managed with intravenous fluids, beta-blockers, and vasoconstrictors to stabilize hemodynamics.

Collectively, these cases highlight the need for vigilant preprocedural risk assessment for latent obstruction and emphasize the importance of rapid recognition and hemodynamic management to prevent life-threatening outcomes following TAVR.

## Conclusion

This case report highlights ventricular suicide as a rare but critical complication following TAVR in a patient with severe AS and HCM. Abrupt afterload reduction led to mid-ventricular systolic collapse, hyperdynamic left ventricular function, and obstructive shock. Prompt recognition and intervention were crucial in stabilizing our patient following hemodynamic collapse post-TAVR. Immediate management with intravenous fluids and phenylephrine effectively restored hemodynamic stability, preventing further deterioration, and beta-blockade was initiated to prevent recurrence.

Given the predisposition of HCM and its phenocopies to adverse outcomes post-TAVR, identifying high-risk patients preoperatively is essential. Preemptive strategies such as echocardiographic assessment for small left ventricular cavity size, preexisting SAM, and latent LVOT obstruction should be incorporated into preprocedural planning. In high-risk patients, prophylactic measures such as preoperative beta-blockade, volume loading, and consideration of temporary pacing to reduce LVOT gradient may prevent post-TAVR hemodynamic collapse and ventricular suicide.

## Learning objectives

- Identification of patients who may present with HCM-like physiology.
- To understand the physiology of obstructive shock in patients post-TAVR procedure.
- Outline the management of shock or “ventricular suicide” in post-TAVR patients.

## Supplementary Information

Additional file 1.

Additional file 2.

Additional file 3.

Additional file 4.

Video 1- Baseline 3 chamber view cine
Additional file 5.

Video 2 - Post TAVR parasternal long-axis view
Additional file 6.

## Data Availability

No datasets were generated or analyzed during the current study.

## Abbreviations

AS: Aortic stenosis
TAVR: Transcatheter aortic valve replacement
LVOT: Left ventricular outflow tract obstruction
EKG: Electrocardiogram
TTE: Transthoracic echocardiography
ASA: Alcohol septal ablation
